# Population pharmacokinetics of dalbavancin: external validation, model averaging, and implications for precision dosing in prolonged therapy

**DOI:** 10.1128/aac.00665-26

**Published:** 2026-06-22

**Authors:** Hamza Sayadi, Matthieu Gregoire, Yeleen Fromage, Marc Labriffe, Franck Maizaud, Caroline Monchaud, Cyrielle Codde, David Boutoille, Pierre Marquet, Jean-Baptiste Woillard

**Affiliations:** 1 Department of Pharmacology, Toxicology and Pharmacovigilance, Dupuytren University Hospital (CHU Dupuytren), Limoges, France; 2 Pharmacology & Transplantation, INSERM U1248, University of Limoges, Limoges, France; 3 Clinical Pharmacology Department, Nantes University, Nantes University Hospital 27045, Nantes, France; 4 Infection and Immunity: Targets and Drugs, IICiMed, UR 1155, Nantes University, Nantes University Hospital 27045, Nantes, France; 5 INSERM CIC 1435, Dupuytren University Hospital (CHU Dupuytren) 36715 , Limoges, France; 6 Department of Infectious and Tropical Diseases, Dupuytren University Hospital (CHU Dupuytren) 36715 , Limoges, France; 7 INSERM, Infectious and Tropical Diseases Department, CIC 1413, Nantes University, Nantes University Hospital 27045, Nantes, France; Providence Portland Medical Center, Portland, Oregon, USA

**Keywords:** gram-positive infection, dalbavancin, population pharmacokinetics, individualized dosing, antimicrobial stewardship

## Abstract

Dalbavancin is increasingly used off-label for prolonged therapy in gram-positive infections, but optimal repeat-dosing strategies remain undefined, and published population pharmacokinetic (popPK) models lack external validation in real-world cohorts. A popPK model was developed using routine therapeutic drug monitoring (TDM) data from 183 adults (406 concentrations) with gram-positive infections and externally validated in an independent cohort (*n* = 30, 92 concentrations). Predictive performance was benchmarked against five published models and a model averaging (MA) approach under three scenarios: a priori prediction and Bayesian forecasting with one or two TDM measurements. Monte Carlo simulations assessed the probability of target attainment (*f*AUC_24_/MIC ≥111.1) for repeat-dose regimens (1,500 mg D1/D8 and D1/D15) at MICs 0.125 and 0.250 mg/L. A two-compartment model with eGFR (clearance) and body weight (clearance and volumes) best described the data. In a priori external validation, it achieved the lowest rRMSE (49.8%) among published models. MA with two TDM measurements yielded optimal performance (rRMSE 26.9%), closely followed by our model (31.4%). Simulations showed that empirical regimens maintained target attainment of ≥90% for 3–7 weeks, heavily dependent on PD targets and patient characteristics. A third 1,500 mg dose, timed to target decline, restored prolonged coverage across all strata. This externally validated popPK model, complemented by MA, provides robust pharmacokinetic foundations for precision dosing in gram-positive infections requiring prolonged therapy. Based on these findings, we propose an investigational sequential precision dosing workflow integrating machine learning with Bayesian forecasting; prospective validation is required before clinical implementation.

## INTRODUCTION

Methicillin-resistant *Staphylococcus aureus* and other complex gram-positive infections remain leading causes of morbidity and mortality, necessitating effective and practical therapeutic alternatives (1, 2). Dalbavancin, a long-acting lipoglycopeptide, exhibits potent activity against these pathogens with a unique pharmacokinetic profile characterized by a prolonged terminal half-life (approximately 14 days), allowing simplified dosing regimens. It is currently approved for acute bacterial skin and skin structure infection (ABSSSI) (3–11).

Beyond this approved indication, dalbavancin’s favorable pharmacokinetic/pharmacodynamic (PK/PD) profile, high tissue penetration, antibiofilm activity, and excellent safety increasingly support its off-label use in complex, deep-seated infections such as osteoarticular infections, infective endocarditis, and prosthetic vascular infections (11–20). These indications frequently require prolonged intravenous antimicrobial therapy. Current practice employs repeated off-label 1,500 mg infusions administered seven or 14 days apart, an approach widely adopted in published real-world cohorts though not formally approved (21–27). However, for patients requiring therapy beyond two doses, optimal re-dosing timing remains undefined.

Therapeutic drug monitoring (TDM) and model-informed precision dosing (MIPD) offer a rational approach to optimize dalbavancin therapy. From a PK/PD perspective, the ratio of the free area under the concentration–time curve to the minimum inhibitory concentration (*f*AUC_24_/MIC) is the primary driver of antibacterial efficacy (28, 29). Recent clinical evidence supports the translational relevance of this target: maintaining dalbavancin concentrations above PK/PD thresholds for ≥70% of treatment duration was associated with a markedly higher probability of clinical success (OR 6.71, 95% CI 0.97–43.3; *P* = 0.05) (30). Similar observational studies in osteoarticular and endovascular infections have shown that sustained target exposure correlates with improved clinical outcomes (31). To achieve these targets consistently, clinicians must account for substantial interindividual variability (IIV) in dalbavancin clearance and exposure, which population pharmacokinetic (popPK) analyses have linked to renal function, body weight (WT), and serum albumin (32–34).

While popPK modeling is essential to quantify interindividual variability and enables target attainment simulation, clinical implementation of existing dalbavancin models faces critical gaps. First, few published models have been externally validated in independent real-world cohorts (32–36), a prerequisite for reliable clinical translation, given that models may exhibit predictive bias when extrapolated to broader populations (37–39). Second, Bayesian TDM-guided approaches require concentration measurements for maximum a posteriori Bayesian estimation (MAP-BE). Although Cojutti et al. demonstrated that two TDM measurements between doses can accurately forecast target attainment duration (40), this sampling strategy may be logistically challenging in routine practice. Our prior work showed that a support vector machine (SVM) algorithm using baseline covariates and a single pre-second-dose concentration outperformed standard MAP-BE for early detection of subtherapeutic exposure (41, 42). These findings suggest a sequential precision-dosing strategy: machine learning (ML) to identify optimal re-dosing timing using early data, followed by popPK-based MAP-BE, potentially enhanced by model averaging (MA), to optimize subsequent doses and intervals by incorporating additional concentrations.

We therefore aimed to (i) develop a dalbavancin popPK model from real-world TDM data, including multidose patients; (ii) externally evaluate it in an independent cohort; (iii) systematically benchmark its predictive performance across a priori and MAP-BE scenarios against five published models and an MA approach; and (iv) use Monte Carlo simulations to estimate the probability of target attainment (PTA) for the two most commonly used off-label repeat-dose schedules (1,500 mg day 1/day 8 [D1/D8] and day 1/day 15 [D1/D15]) across clinically relevant patient subgroups.

## MATERIALS AND METHODS

### Study design and data source

We performed a retrospective observational popPK study using routine TDM data from de-identified adult patients treated with dalbavancin at Limoges University Hospital between March 2021 and March 2026. Eligible patients had received ≥1 dalbavancin dose and had ≥1 plasma concentration measured as part of routine care. Blood samples were collected according to routine clinical practice, predominantly at trough (pre-dose) and during the late elimination phase. This pragmatic design applied no exclusion criteria for dosing regimens or sampling schedules to capture real-world heterogeneity.

Collected data included age, sex, WT, height, body mass index (BMI), serum creatinine, estimated glomerular filtration rate (eGFR, estimated using the CKD-EPI equation (43), clinical indication, microbiological results, and detailed administration history (dose, date, duration, and infusion time). Serum albumin was not systematically available in this real-world cohort and could not be evaluated as a covariate.

External validation was performed in an independent clinical cohort (Nantes University Hospital) with no data overlap or parameter updating from the development cohort.

### Bioanalytical methods

Blood samples were collected in EDTA tubes and centrifuged immediately upon receipt in the laboratory. Plasma was stored at −20°C until analysis. Samples from external centers were shipped frozen on dry ice. Total plasma dalbavancin concentrations in both cohorts were quantified using validated liquid chromatography–tandem mass spectrometry (44). The Limoges assay was linear up to 100 mg/L with a lower limit of quantification (LLOQ) of 0.5 mg/L, while the Nantes assay was linear up to 200 mg/L with an LLOQ of 1 mg/L. The different LLOQs reflected method validation protocols at each site but did not impact model development, as all measured concentrations exceeded both LLOQs. Samples exceeding calibration ranges were diluted 1:10 and re-assayed with validated dilution integrity. Both laboratories participated in the external quality assessment (Asqualab, Paris, France) with consistently satisfactory performance (*Z*-scores <2) since 2021.

### Population pharmacokinetic modeling

#### Structural model development

Analyses were performed using non-linear mixed-effect modeling with the Stochastic approximation expectation–maximization algorithm implemented in Monolix (v2024R1; Lixoft, Antony, France). Structural models with one, two, or three compartments with linear elimination and intravenous infusion input were evaluated. Infusion durations were fixed to observed values or set to a default of 30 min when missing. IIV was modeled exponentially on relevant PK parameters (clearance [CL], central volume of distribution [*V1*], intercompartmental clearance [*Q*], and peripheral volume [*V2*]). Residual unexplained variability (RUV) was evaluated using additive, proportional, or combined error models. Model selection was based on the Bayesian information criterion (BIC), parameter precision (relative standard error [RSE] <30%), and standard diagnostic plots (observed vs predicted, individual fits, individual weighted residuals [IWRES], and normalized prediction distribution errors [NPDE]).

#### Covariate analysis

Candidate covariates included age, sex, WT, BMI, serum creatinine, and eGFR. Missing baseline values were imputed using k-nearest neighbors (KNN, *k* = 5, VIM package in R) (45). Covariate selection followed a stepwise forward inclusion (*P* < 0.05, ΔOFV ≥3.84) and backward elimination (*P* < 0.01, ΔOFV ≥6.63) procedure. Continuous covariates were centered on their population median and tested using power functions (allometric scaling):


$$log\theta_{i}=\;log\theta_{pop}+\;log\theta_{cov}\times \left(\frac{{Cov}_{i}}{{Cov}_{median}}\right).$$

Categorical covariates were modeled as fractional changes on the typical parameter value:


$$\theta j=\;\theta_{jTV}\;\times \;e{\;}^{\theta Covi\;\times Covi}.$$

#### Model evaluation

Final model performance was evaluated using parameter precision, goodness-of-fit plots, NPDEs, and prediction-corrected visual predictive checks (pcVPC). Non-parametric bootstrap resampling (1,000 replicates) assessed parameter uncertainty and model robustness.

### External evaluation and model comparison

Five published dalbavancin popPK models (Carrothers et al. [32], Cojutti et al. [33, 35], Chiriac et al. [36], and Baiardi et al. [34]) were re-implemented in R using the mrgsolve package (46). These five models, alongside the developed model, were applied to the external evaluation cohort to benchmark their predictive performance. Additionally, an MA approach encompassing all six models was constructed and evaluated on the same data set. Model specifications are summarized in Table 1.

**TABLE 1:** Comparative analysis of published population pharmacokinetic models of dalbavancin^*a*^^*,**b*^

Study (publication, year)	Number of subjects	Number of observations	Patient characteristics	Structural model	Covariate	Estimation and accuracy (%) of population parameters	Between-subject variability (%)	Residual unexplained variability (%)
Carrothers et al. (Clin Pharmacol Drug Dev., 2020) (32)	703	2,307	ABSSSI, catheter-related bloodstream infections	Three compartments, zero-order input, first-order elimination	CRCL and WT on CL, WT and ALB on *V1*, ALB and age on *V2*, WT and ALB on *V3*	CL: 0.0531 (1.1) *V1*: 3.04 (4.1) *Q2*: 0.288 (13.2) *V2*: 8.78 (3.9) *Q3*: 2.11 (10.8) *V3*: 3.28 (9.6)	CL: 22.4 *V1*: 40.7 *V2*: 34.3 *V3*: 74.0	Proportional: 19.2
Cojutti et al. (Antimicrob Agents Chemother., 2023) (33)	15	120	Staphylococcalbone and joint infections	Two compartments, zero-order input, first-order elimination	No covariate retained	CL: 0.106 (9.33) *V1*: 17.40 (12.0) *Q*: 0.103 (37.4) *V2*: 15.10 (15.8)	CL: 36.21 *V1*: 44.27 *Q*: 130.46 *V2*: 62.34	Proportional: 18.0
Cojutti et al. (Antibiotics, 2023) (35)	69	289	Bone and joint infections, endocarditis and/or prosthetic endovascular infections	Two compartments, zero-order input, first-order elimination	CRCL on CL	CL: 0.029 (11.6) *V1*: 6.14 (5.26) *Q*: 0.026 (18.1) *V2*: 9.52 (19.0)	CL: 26.44 *V1*: 16.10 *Q*: 50.90 *V2*: 37.15	Proportional: 33.92
Chiriac et al. (Antibiotics, 2024) (36)	13	39	Ventricular assist device infections	Two compartments, zero-order input, first-order elimination	No covariate retained	CL: 0.05 (6.83) *V1*: 6.5 (8.5) *Q*: 0.476 (fixed) *V2*: 15.4 (12.3)	CL: 50.9 *V1*: 54.5 *V2*: 71.2	Proportional: 10.0
Baiardi et al. (Antibiotics, 2025) (34)	30	190	ABSSSI, bone and joint infections, endocarditis and/or prosthetic endovascular infections, prostatic abscess	Two compartments, zero-order input, first-order elimination	WT on CL, *Q*, *V1*, and *V2*	CL: 0.0273 (5.1) *V1*: 3.6 (3.7) *Q*: 0.0225 (28.4) *V2*: 6.4 (11.9)	CL: 22.0 *V1*: 17.3 *Q*: 55.9 *V2*: 30.1	Proportional: 14.4

^*a*^ Parameters are reported as typical population values with relative standard error in parentheses. Between-subject variability is expressed as the coefficient of variation (CV) (CV%). Residual unexplained variability is reported as proportional error.

^*b*^ ABSSSI, acute bacterial skin and skin structure infection; ALB, albumin; CL, clearance; CRCL, creatinine clearance; *Q*, intercompartmental clearance; *V1*, central volume of distribution; *V2*, peripheral volume of distribution; *V3*, second peripheral volume of distribution; WT, body weight.

Three scenarios were evaluated: (i) a priori predictions (no individual TDM information) (population parameters fixed to published estimates, IIV terms set to zero, predictions generated from dosing history, sampling times, and covariates only); (ii) MAP-BE using one TDM measurement (parameters updated using the first concentration [mapbayr package]) (47); and (iii) MAP-BE using two TDM measurements. For MA, individual MAP-BE estimation was performed independently with all six models using the observed TDM concentrations. Model-specific weights were calculated based on individual log-likelihoods, and the final prediction at each time point was computed as the weighted average across models (detailed in the supplemental material). This approach accounts for model uncertainty by proportionally weighting predictions according to each model’s goodness of fit to the individual’s TDM data (48).

Predictive performance metrics included median prediction error (MDPE) for bias; median absolute prediction error (MDAPE) for imprecision; f20 and f30 metrics (percentages of predictions within ±20% and ±30% of observed concentrations, respectively); relative root mean square error (rRMSE) as a global error metric; and Lin’s concordance correlation coefficient (CCC), decomposed into the Pearson correlation coefficient and Lin’s bias correction factor. Pairwise comparisons against the developed model were performed using paired Wilcoxon signed-rank tests on individual MDPE and MDAPE values, with *P* values adjusted for multiple comparisons using the Holm method. *P* < 0.05 was considered statistically significant. All analyses were performed in R (v4.5.2).

### Monte Carlo simulations and probability of target attainment

Monte Carlo simulations were performed in R using the mrgsolve package (46). Virtual populations (*n* = 10,000 per stratum) were simulated by applying the estimated population parameters, IIV, and RUV to covariate profiles. Two dosing schedules were evaluated: 1,500 mg on D1/D8 and 1,500 mg on D1/D15. A hypothetical third 1,500 mg dose was simulated at the time point when PTA fell below 90% within each stratum, computed both as a population mean and individually for each simulated patient. The 95% CI for the critical day was derived from the 2.5th and 97.5th percentiles of individual distribution. Simulations were followed for 100 days.

The primary PK/PD target was *f*AUC_24_/MIC ≥111.1, validated in murine models and associated with a 2-log bacterial reduction against *Staphylococcus aureus*. (28, 29) Simulated total concentrations were converted to unbound concentrations assuming a fraction unbound (fu) of 0.07 (4). Unbound AUC_24_ was computed by numerical trapezoidal integration (pracma package). Two MIC analyses were performed: 0.250 mg/L (EUCAST v15.0 clinical breakpoint for *Enterococcus faecium*, representing a conservative scenario) and 0.125 mg/L (EUCAST v15.0 clinical breakpoint for *Staphylococcus aureus*) (49). These values represent the upper limits of the susceptible range, ensuring a conservative, worst-case scenario for target attainment.

## RESULTS

### Pharmacokinetic modeling results

#### Study population

The popPK analysis data set comprised 183 patients contributing 406 dalbavancin plasma concentrations, all of which were above the LLOQ. Individual concentration–time profiles are provided in Fig. S1, and baseline demographics, dosing history, treatment indications, and identified pathogens are summarized in Table 2. The development cohort was elderly (median age 77 years, IQR: 67–83, range: 23–99) and predominantly male (62.8%). Renal function was highly heterogeneous (median eGFR 75 mL/min/1.73 m², IQR: 49–87, range: 14–135), and WT varied widely (median 79 kg, IQR: 65–88, range: 32–138). Patients received between 1 and 23 dalbavancin doses, with 1–25 TDM samples collected per patient. Notably, about a quarter of the cohort underwent prolonged therapy (≥3 doses, 24.6%) and intensive monitoring (≥3 TDM samples, 25.1%). The most frequent indications were osteoarticular infections (43.2%) and infective endocarditis (12.6%). When microbiological documentation was available, staphylococcal species predominated (*Staphylococcus aureus*, 19.7%; coagulase-negative staphylococci, 30.1%). Missing baseline covariates were observed for height (36 patients, 19.7%), weight (27 patients, 14.8%), and serum creatinine (29 patients, 15.8%), and were imputed using the pre-specified KNN approach.

**TABLE 2:** Demographic and clinical characteristics of the study population (*N* = 183)*^c^*^*,*^*^d^*

Characteristic	Value
Demographics	
Age (years), median (IQR)	77 (67–83)
Sex, *n* (%)	
Male	115 (62.8)
Female	68 (37.2)
Body weight (kg), median (IQR)	79 (65–88)
Height (cm), median (IQR)	170 (163–178)
Body mass index (kg/m²), median (IQR)	25 (23–30)
Renal function	
Serum creatinine (µmol/L), median (IQR)	87 (65–110)
eGFR (mL/min/1.73 m²), median (IQR)	75 (49–87)
Treatment characteristics	
Total dalbavancin doses per patient, *n* (%)	
1	87 (47.5)
2	51 (27.9)
3	21 (11.5)
≥4	24 (13.1)
TDM samples per patient, *n* (%)	
1	93 (50.8)
2	44 (24.0)
3	17 (9.3)
≥4	29 (15.8)
Clinical indication, *n* (%)	
Osteoarticular infection	79 (43.2)
Infective endocarditis	23 (12.6)
Vascular prosthetic infection	11 (6.0)
Bacteremia	4 (2.2)
ABSSSI	2 (1.1)
Other/not specified	64 (35.0)
Identified pathogen, *n* (%)	
Coagulase-negative staphylococci*^a^*	55 (30.1)
*Staphylococcus aureus*	36 (19.7)
*Streptococcus* spp.*^b^*	3 (1.6)
*Enterococcus faecalis*	7 (3.8)
*Enterococcus faecium*	6 (3.3)
*Corynebacterium* spp.	2 (1.1)
No pathogen identified/not available	74 (40.4)

^*a*^ Includes *Staphylococcus epidermidis* (*n* = 53), *Staphylococcus haemolyticus* (*n* = 1), and *Staphylococcus capitis* (*n* = 1).

^*b*^ Includes *Streptococcus dysgalactiae* (*n* = 2) and *Streptococcus parasanguinis* (*n* = 1).

^*c*^ ABSSSI, acute bacterial skin and skin structure infection; eGFR, estimated glomerular filtration rate (CKD-EPI equation); IQR, interquartile range;TDM, therapeutic drug monitoring.

^*d*^ Missing data: serum creatinine (*n* = 29), eGFR (*n* = 29), weight (*n* = 27), height (*n* = 36).

The external evaluation cohort comprised an independent clinical data set of 30 adult patients treated at Nantes University Hospital, contributing a total of 92 plasma concentrations. The cohort was younger (median 63 vs 77 years) with better renal function (median eGFR 88 vs 75 mL/min/1.73 m²) than the development cohort, while body weight (75 vs 79 kg), sex distribution (70% vs 63% male), and BMI (25 vs 25 kg/m²) were comparable. Specifically, the median age was 63 years (IQR: 46–72, range: 16–90); the median WT was 75 kg (IQR: 60–90, range: 40–105); and the median eGFR was 88 mL/min/1.73 m² (IQR: 61–111, range: 13–161). Patients received between 1 and 7 dalbavancin doses, with one to seven TDM samples collected per patient. Notably, a substantial proportion of these evaluation patients underwent prolonged therapy and intensive monitoring, with 36.6% receiving three or more dalbavancin doses and 46.6% contributing three or more TDM samples. Complete baseline characteristics for the external evaluation cohort are summarized in Table 3.

**TABLE 3:** Demographic and clinical characteristics of the external validation cohort (*N* = 30)^*a*^

Characteristics	Value
Demographics	
Age (years), median (IQR)	63 (46–72)
Sex, *n* (%)	
Male	21 (70)
Female	9 (30)
Body weight (kg), median (IQR)	75 (60–90)
Height (cm), median (IQR)	171 (160–173)
Body mass index (kg/m²), median (IQR)	25.0 (22–29)
Renal function	
Serum creatinine (µmol/L), median (IQR)	80 (65–100)
eGFR (mL/min/1.73 m²), median (IQR)	88 (61–111)
Treatment characteristics	
Total dalbavancin doses per patient, *n* (%)	
1	11 (36.7)
2	8 (26.7)
3	4 (13.3)
≥4	7 (23.3)
TDM samples per patient, *n* (%)	
1	10 (33.3)
2	6 (20.0)
3	4 (13.3)
≥4	10 (33.3)

^*a*^ eGFR, estimated glomerular filtration rate (CKD-EPI equation); IQR, interquartile range;TDM, therapeutic drug monitoring.

#### Structural model and covariates

A two-compartment model with first-order elimination best described the data (BIC = 2,981.7) compared with one-compartment (BIC = 3,193.6) and three-compartment (BIC = 2,986.9) structures. IIV was supported on *CL*, *V1*, *Q*, and *V2*; RUV was best described by a proportional error model.

Covariate analysis identified eGFR and WT as statistically significant predictors of dalbavancin *CL*. Inclusion of eGFR on *CL* significantly improved model fit (ΔOFV = −14.1) and reduced IIV on CL from 24.0% to 21.3%; subsequent inclusion of WT on *CL* further improved the model (ΔOFV = −38.8) and reduced IIV on *CL* to 17.5%.

Body weight also significantly influenced *V1* and *V2* (ΔOFV = −11.16, df = 2, *P* < 0.01). However, free estimation of allometric exponents for *V1* and *V2* yielded imprecise values (RSE 47%–53%) with confidence intervals overlapping 1.0. Fixing these exponents to 1.0, consistent with standard allometric scaling for distribution volumes (50, 51), improved parsimony (ΔBIC = −5.01) and parameter precision (all RSE <25%) without significantly degrading model fit (likelihood ratio test: ΔOFV = +5.40, df = 2, *P* = 0.067). This approach maintained the beneficial effect of body weight on distribution volumes while ensuring model stability. No other candidate covariates met the required statistical criteria.

#### Parameter estimates and diagnostics

Final parameter estimates and bootstrap confidence intervals are reported in Table 4. All fixed-effect parameters were estimated with acceptable precision (RSE <25%). Typical population *CL* was 0.035 L/h (RSE 3.0%), with WT (*β* = 0.61, RSE 12.7%) and eGFR (*β* = 0.22, RSE 19.9%) as significant covariates. Typical *V1* was 4.74 L (RSE 4.9%), and the *V2* was 8.74 L (RSE 7.1%). Bootstrap resampling (1,000 replicates, 100% convergence) confirmed parameter estimates with bias ranging from −7.0% (βWT,*CL*) to +19.9% (*Q*). IIV ranged from moderate to high (coefficients of variation 17.5%–52.4%), with the lowest variability observed for clearance (CV 17.5%, RSE 10.4%) and the highest for *Q* (CV 52.4%, RSE 23.5%). Bootstrap recovery of variability parameters was excellent (bias from −12.1% to −1.2%). Shrinkage was moderate for *CL* (27.7%) but substantial for *Q* (47.6%), *V2* (51.4%), and *V1* (75.7%), consistent with the sparse and predominantly late sampling strategy inherent to real-world dalbavancin TDM. The proportional residual error was estimated at 21% (RSE 5.3%, 95% CI 19%–23%).

**TABLE 4:** Population pharmacokinetic parameters for dalbavancin: final model estimates and bootstrap results^*a*^^,^^*b*^

Parameters	Value		Stochastic approximation				Bootstrap (*N* = 1,000)		
			SE	RSE (%)	P2.5	P97.5	P2.5	P50.0	P97.5
Fixed effects									
TV_Cl	0.035		0.001	2.99	0.033	0.037	0.031	0.035	0.04
βeGFR,CL	0.22		0.043	19.92	0.13	0.3	0.11	0.21	0.32
βWT,CL	0.61		0.077	12.67	0.45	0.76	0.4	0.56	0.74
TV_V1	4.74		0.23	4.86	4.31	5.21	3.93	4.58	5.51
βWT,V1	1								
TV_Q	0.033		0.0045	13.66	0.025	0.042	0.023	0.037	0.068
TV_V2	8.74		0.62	7.12	7.61	10.04	7.68	9.25	11.56
βWT,V2	1								
Standard deviation of the random effects									
	Value	CV%							
ω CL	0.17	17.5	0.018	10.37	0.14	0.21	0.098	0.17	0.24
ω *V1*	0.2	20.02	0.046	23.4	0.13	0.31	0.098	0.18	0.33
ω *Q*	0.49	52.39	0.12	23.54	0.32	0.77	0.24	0.43	0.65
ω *V2*	0.34	35.22	0.058	16.95	0.25	0.47	0.18	0.31	0.46
Error model parameters									
σ prop	0.21		0.011	5.31	0.19	0.23	0.18	0.21	0.23

^*a*^ Parameter estimates are presented with standard errors (SEs), relative standard errors (RSEs), and 95% confidence intervals from non-parametric bootstrap resampling (1,000 replicates with replacement).Weight exponents on V1 and V2 (βWT,V1 and βWT,V2) were fixed to 1.0. P2.5, P50.0, and P97.5 represent the 2.5th, 50th (median), and 97.5th percentiles of the bootstrap distribution, respectively.

^*b*^ CL, clearance (L/h); Q, intercompartmental clearance (L/h); TV, typical population value; *V1*, central volume of distribution (L); *V2*, peripheral volume of distribution (L); β, covariate effect (power exponent); ω, inter-individual variability (standard deviation, with corresponding coefficient of variation [CV%]).

**Fig 1:**
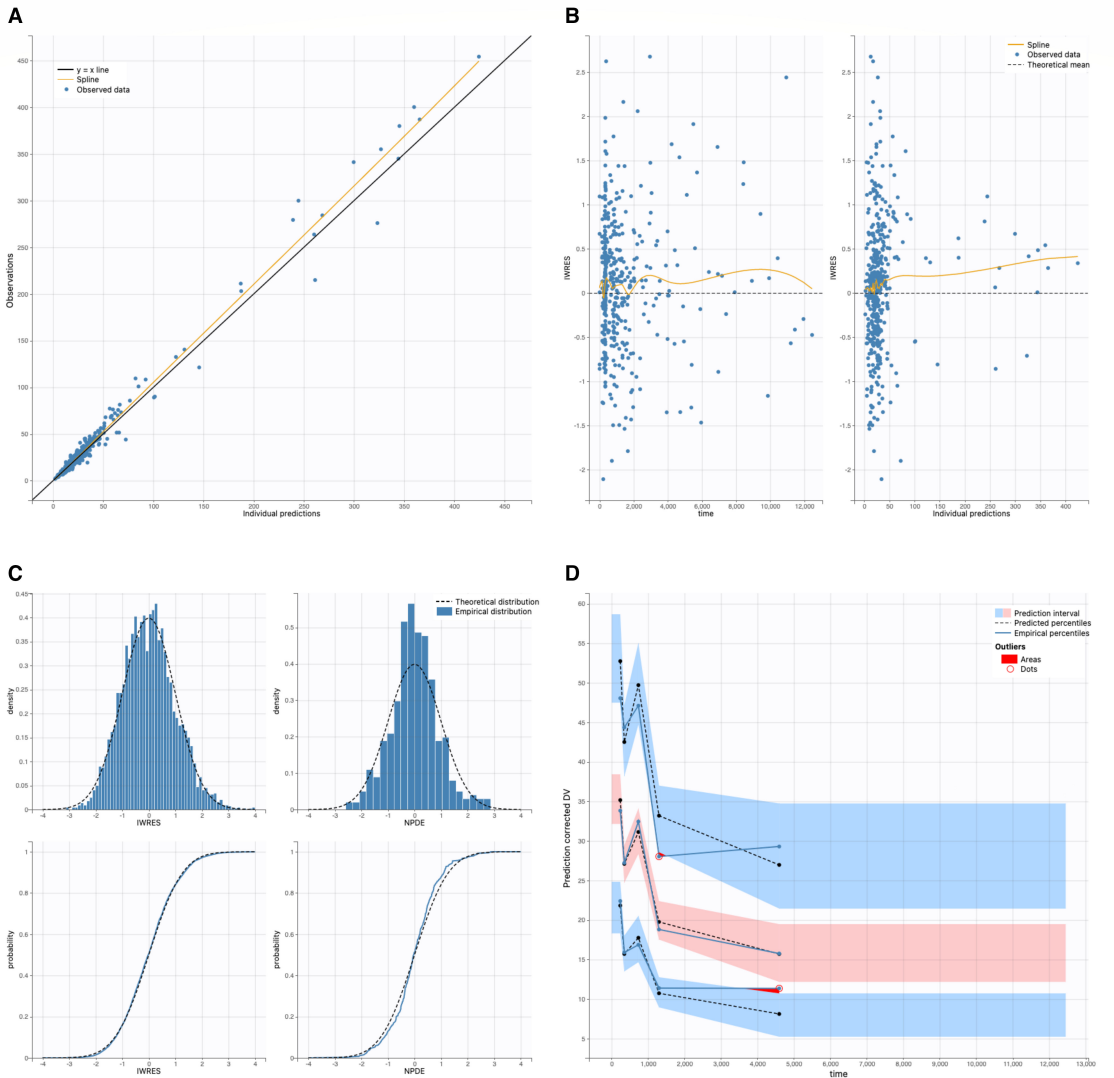
Diagnostic plots and prediction-corrected visual predictive check for the final population pharmacokinetic model. (**A**) Observed versus individual predicted concentrations.(**B**) Individual weighted residuals (IWRES) versus time (left panel) and versus individual predictions (right panel). (**C**) Distribution and quantile–quantile assessment of IWRES (left panels) and normalized prediction distribution errors (NPDE) (right panels). (**D**) Prediction-corrected visual predictive check (pcVPC) comparing observed and model-predicted concentration percentiles over time.

Goodness-of-fit plots showed excellent agreement between observed and individual predicted concentrations across the entire concentration range (1–450 mg/L), with data points evenly distributed around the line of identity (Fig. 1A). IWRES were symmetrically distributed around zero with no trend vs time or predictions (Fig. 1B). Empirical distributions of IWRES and NPDE closely matched the theoretical standard normal distribution (Fig. 1C). The pcVPC demonstrated adequate predictive performance, with observed 10th, 50th, and 90th percentiles falling within 95% prediction intervals of corresponding simulated percentiles across the full concentration–time range (Fig. 1D). Additional diagnostics confirmed model adequacy (Fig. S2).

### External validation and model comparison

In external validation without TDM information, a priori predictions were suboptimal for all models (rRMSE range 49.8–115.8%; Table 5; Fig. S3). The developed model achieved the lowest rRMSE (49.8%) with balanced bias and precision (MDPE 20.7%, MDAPE 25.9%, CCC 0.930), though differences vs Carrothers et al. (32) (52.7%) and Cojutti et al. (35) (56.5%) were not statistically significant (both *P* > 0.05). The Cojutti et al. model exhibited severe systematic bias (MDPE −54.6%, *P* < 0.0001, CCC 0.386) (33).

**TABLE 5:** External validation and comparative predictive performance of seven population pharmacokinetic approaches for dalbavancin in the Nantes cohort*^a^*^,^^*b*^^*,*^*^c^*

Model	*n*	MDPE (IQR) (%)	MDAPE (IQR) (%)	f20 (%)	f30 (%)	rRMSE (%)	CCC (Cb)
A priori prediction (no TDM informatio*n*)							
Developed model	92	20.7 (−3.4 to 52.6), ref.	25.9 (12.4 to 52.6), ref.	40.2	54.3	49.8	0.930 (0.99)
Carrothers et al. (32)	92	19.1 (−10.5 to 49.0), ns	27.0 (16.0 to 53.6), ns	33.7	54.3	52.7	0.916 (1.00)
Cojutti et al. (35)	92	−5.0 (−30.2 to 15.2)****	24.9 (9.8 to 41.1), ns	43.5	56.5	56.5	0.884 (0.97)
Chiriac et al. (36)	92	14.9 (−11.3 to 57.7), ns	37.5 (13.6 to 60.9), ns	32.6	39.1	63.2	0.832 (0.92)
Baiardi et al. (34)	92	51.1 (13.9 to 70.1)****	51.1 (15.8 to 70.1)****	29.3	32.6	81.8	0.863 (0.92)
Cojutti et al. (33)	92	−54.6 (−66.3 to −41.4)****	55.6 (41.9 to 67.3), ns	4.3	8.7	115.8	0.386 (0.42)
MAP-BE with one TDM measurement							
Model averaging	62	3.8 (−15.2 to 26.3)**	19.6 (9.5 to 32.1), ns	51.6	74.2	30.4	0.963 (0.99)
Developed model	62	12.4 (−12.3 to 35.3), ref.	22.3 (12.5 to 38.5), ref.	45.2	64.5	34.0	0.952 (0.98)
Baiardi et al. (34)	62	10.2 (−4.4 to 43.3), ns	22.4 (9.9 to 43.3), ns	48.4	59.7	35.3	0.960 (0.99)
Carrothers et al. (32)	62	−6.2 (−23.6 to 23.0)*	23.6 (14.1 to 35.9), ns	41.9	62.9	36.2	0.948 (0.99)
Chiriac et al. (36)	62	4.6 (−22.4 to 40.6), ns	30.3 (10.5 to 52.1), ns	37.1	50.0	55.1	0.849 (0.90)
Cojutti et al. (35)	62	−6.7 (−26.7 to 11.7)****	21.2 (10.6 to 41.0), ns	48.4	62.9	63.8	0.797 (0.88)
Cojutti et al. (33)	62	−29.2 (−54.8 to −0.9)****	34.8 (18.1 to 57.9), ns	27.4	40.3	91.5	0.536 (0.60)
MAP-BE with two TDM measurements							
Model averaging	42	−7.6 (−14.9 to 8.5)***	13.9 (7.9 to 35.1), ns	64.3	69.0	26.9	0.971 (1.00)
Developed model	42	2.9 (−14.1 to 20.9), ref.	17.3 (6.3 to 27.6), ref.	59.5	78.6	31.4	0.959 (0.99)
Chiriac et al. (36)	42	−8.1 (−37.9 to 3.8)*	18.2 (5.2 to 49.9), ns	52.4	54.8	34.8	0.950 (0.99)
Baiardi et al. (34)	42	−1.4 (−8.9 to 23.3), ns	19.4 (8.2 to 42.4), ns	52.4	66.7	34.8	0.957 (1.00)
Carrothers et al. (32)	42	−4.4 (−19.2 to 11.5)*	17.1 (7.8 to 36.2), ns	57.1	66.7	36.1	0.951 (1.00)
Cojutti et al. (35)	42	−11.7 (−31.9 to 8.2)**	25.1 (10.9 to 37.3), ns	40.5	59.5	59.8	0.818 (0.90)
Cojutti et al. (33)	42	−26.0 (−47.4 to −7.9)***	36.1 (19.4 to 52.6), ns	26.2	45.2	66.3	0.771 (0.82)

^*a*^ Models are sorted within each scenario by ascending relative root mean square error (rRMSE). The developed model refers to the population pharmacokinetic model developed in this work and serves as the reference for all statistical comparisons. “Model averaging” combines the six published models using Bayesian model averaging based on individual log-likelihoods.

^*b*^ Statistical significance: *P* values from paired Wilcoxon signed-rank tests on individual MDPE (bias) and MDAPE (precision) vs the developed model, adjusted for multiple comparisons. *P < 0.05, **P < 0.01, ***P < 0.001, ****P < 0.0001; ns, not significant (*P* > 0.05); ref., reference model.

^*c*^ Cb, Lin's bias correction factor; CCC, Lin's concordance correlation coefficient; f20, percentage of predictions within ±20% of the observed concentration; f30, percentage of predictions within ±30% of the observed concentration; IQR, interquartile range; MAP-BE, maximum a posteriori Bayesian estimation; MDAPE, median absolute prediction error; MDPE, median prediction error; *n*, number of evaluated concentrations; rRMSE, relative root mean square error; TDM, therapeutic drug monitoring.

With one TDM measurement, MA achieved the best performance (rRMSE 30.4%, CCC 0.963; Fig. S4), closely followed by the developed model (rRMSE 34.0%, CCC 0.952) and Baiardi et al. (35.3%, *P* > 0.05 vs the developed model) (34). With two TDM measurements, MA further improved (rRMSE 26.9%, CCC 0.971; Fig. S5), with the developed model ranking second (rRMSE 31.4%, CCC 0.959). Most published models converged to comparable precision (rRMSE 34.8–36.1%, all *P* > 0.05), except Cojutti et al., which retained substantial bias (MDPE −26.0%, rRMSE 66.3%) (33).

### Monte Carlo simulations and probability of target attainment

To isolate covariate effects, simulations were stratified by (i) fixed median WT with varying eGFR (15–29, 30–59, 60–89, and 90–120 mL/min/1.73 m²) and (ii) fixed median eGFR with varying WT (30–49, 50–79, 8–119, and120–200 kg).

**Fig 2:**
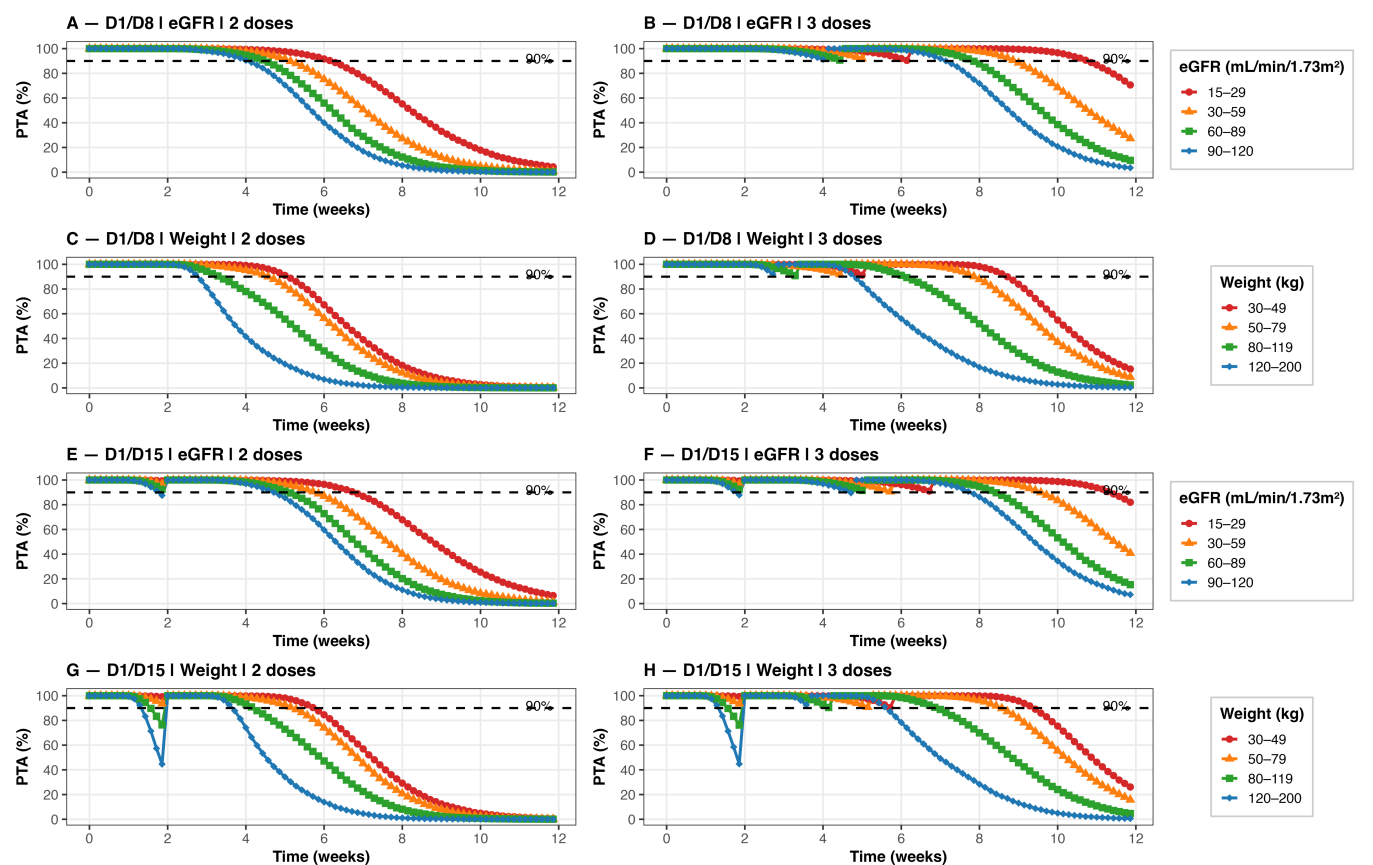
Simulated probability of target attainment (PTA) over time at an MIC of 0.250 mg/L, stratified by renal function and body weight. (**A**) Dalbavancin administered on days 1 and 8 (D1/D8 regimen) , PTA according to eGFR, 2-dose schedule. (**B**) D1/D8 regimen, PTA according to eGFR, 3-dose schedule. (**C**) D1/D8 regimen, PTA according to body weight, 2-dose schedule. (**D**) D1/D8 regimen, PTA according to body weight, 3-dose schedule. (**E**) Dalbavancin administered on days 1 and 15 (D1/D15 regimen), PTA according to eGFR, 2-dose schedule. (**F**) D1/D15 regimen, PTA according to eGFR, 3-dose schedule. (**G**) D1/D15 regimen, PTA according to body weight, 2-dose schedule. (**H**) D1/D15 regimen, PTA according to body weight, 3-dose schedule.

At a MIC of 0.250 mg/L, both eGFR and WT substantially influenced PTA duration. Under D1/D8, PTA ≥90% was maintained for 29–44 days (median across eGFR strata, individual variability 95% CI 22–87 days), with patients having lower eGFR achieving longer coverage. A third 1,500 mg dose extended coverage to 50–76 days (95% CI 45–100) (Fig. 2A and B). The D1/D15 regimen provided slightly longer initial coverage (34–48 days, 95% CI 28–91), with a third dose extending PTA to 55–79 days (95% CI 50–100) (Fig. 2E and F).

Body weight showed similarly marked effects: under D1/D8, PTA ≥90% persisted for 20–36 days (95% CI 18–70) across WT strata, with heavier patients requiring earlier re-dosing. A third dose extended coverage to 34–61 days (95% CI 31–97) (Fig. 2C and D). For D1/D15, initial coverage ranged from 26 to 41 days (95% CI 24–74), extending to 40–66 days (95% CI 37–100) after a third dose (Fig. 2G and H). Notably, in patients ≥80 kg under D1/D15, simulated PTA dropped below 90% before the planned second dose at day 15 (critical days: 12 and 7 for 80–119 kg and 120–200 kg strata, respectively). While the scheduled day 15 dose temporarily restored target attainment, this transient gap suggests D1/D15 may be less reliable for heavier patients treating less susceptible pathogens, potentially warranting D1/D8 regimen or tailored loading strategies.

**Fig 3:**
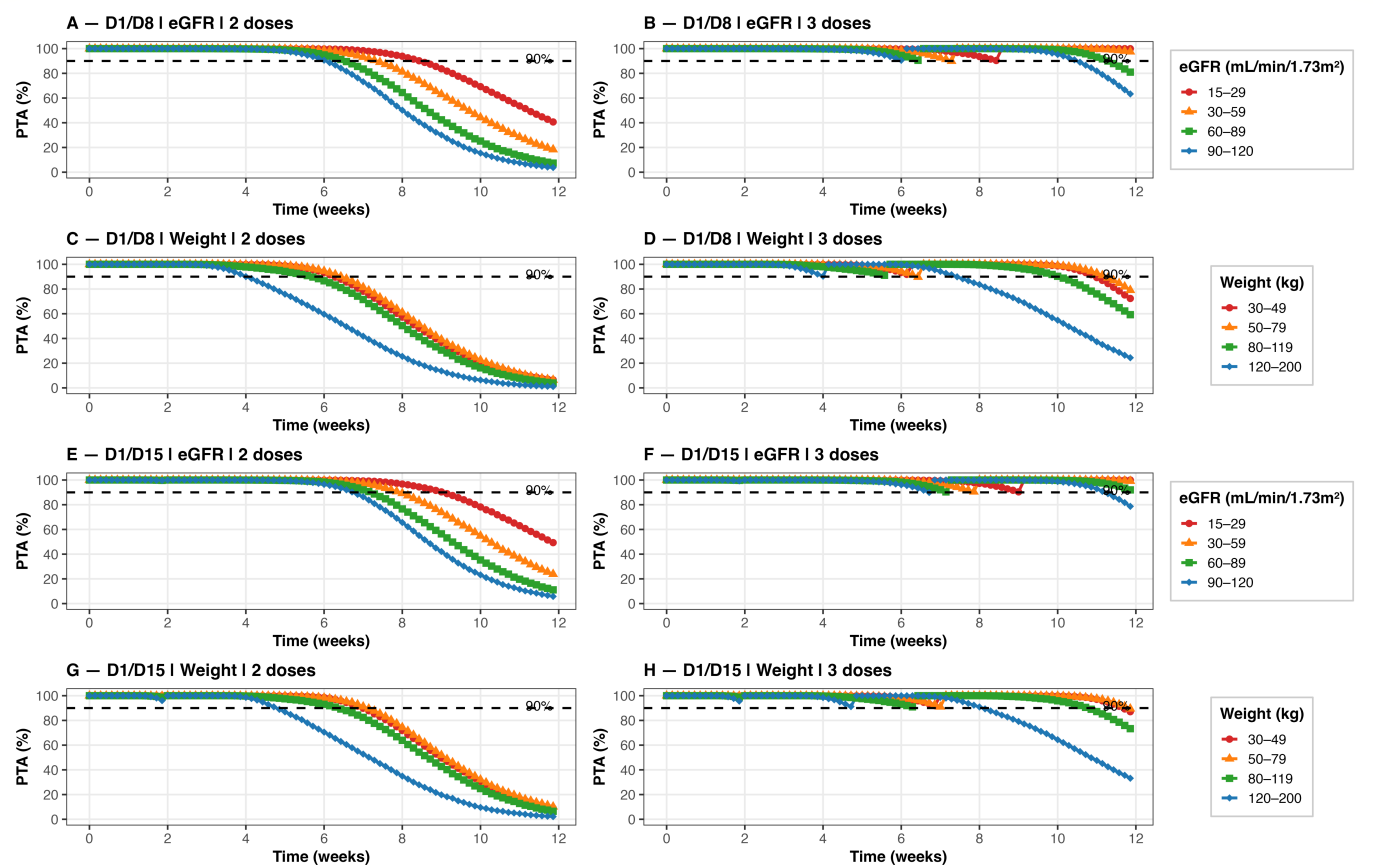
Simulated probability of target attainment (PTA) over time at an MIC of 0.125 mg/L, stratified by renal function and body weight. (**A**) Dalbavancin administered on days 1 and 8 (D1/D8 regimen) , PTA according to eGFR, 2-dose schedule. (**B**) D1/D8 regimen, PTA according to eGFR, 3-dose schedule. (**C**) D1/D8 regimen, PTA according to body weight, 2-dose schedule. (**D**) D1/D8 regimen, PTA according to body weight, 3-dose schedule. (**E**) Dalbavancin administered on days 1 and 15 (D1/D15 regimen), PTA according to eGFR, 2-dose schedule. (**F**) D1/D15 regimen, PTA according to eGFR, 3-dose schedule. (**G**) D1/D15 regimen, PTA according to body weight, 2-dose schedule. (**H**) D1/D15 regimen, PTA according to body weight, 3-dose schedule.

At an MIC of 0.125 mg/L (Fig. 3; Table S1), target attainment was longer overall but the same patterns of interindividual variability persisted. Higher eGFR and greater WT consistently shortened effective coverage duration. Timely administration of a third 1,500 mg dose, guided by model-based predictions, restored prolonged target attainment across all strata, supporting MIPD for extended therapy.

## DISCUSSION

We developed and externally validated a two-compartment population pharmacokinetic model for dalbavancin using a large, heterogeneous, real-world population (*n* = 183, 406 concentrations). The final model incorporated eGFR as a covariate on clearance and body weight on both clearance and volumes of distribution. This two-compartment structure reflects dalbavancin’s distribution phase and aligns with most previously published models (33–36). Although Carrothers et al. (32) described a three-compartment model, this discrepancy is likely attributable to a lack of systematic early post-infusion sampling in our cohort.

Both eGFR (*β* = 0.22) and WT (*β* = 0.61) significantly influenced clearance, consistent with mixed elimination pathways (4). The body weight exponent on *CL* (0.61) was lower than the theoretical allometric value of 0.75. This subproportional scaling may reflect body composition differences in this elderly population (median age 77 years), age-related changes in organ function, or residual correlation with eGFR. From a dosing perspective, this finding suggests weight-based adjustments should account for this subproportional relationship. Body weight also influenced *V1* and *V2*, consistent with physiological expectations that distribution volumes scale with body size. Fixing allometric exponents to 1.0 improved parsimony while preserving the beneficial effect of body weight on volumes and ensuring model stability for clinical applications (50, 51).

Independent external validation assesses model generalizability and predictive performance in independent populations, a prerequisite for confident clinical translation (37–39). Despite demographic differences, our developed model achieved the lowest a priori rRMSE among all evaluated approaches. Overall, a priori predictions proved insufficient for precision dosing. Notably, the covariate-free Cojutti et al. model (33) exhibited severe underprediction, reflecting an inflated typical clearance (0.106 L/h) derived from a small cohort (*n* = 15). Although our validation sample (*n* = 30) limits definitive superiority claims, this finding underscores that population-level estimates cannot reliably predict individual exposure without TDM data. MAP-BE substantially improved performance for most models. With one TDM measurement, rRMSE decreased across models via empirical Bayes shrinkage, with varying degrees of improvement depending on model characteristics. For instance, the Baiardi et al. model (34), despite marked a priori overprediction (MDPE, +51.1%), achieved a predictive accuracy (rRMSE, 35.3%) comparable to our developed model (34.0%, *P* > 0.05) after incorporating a single concentration. This example illustrates that Bayesian updating with individual observations can substantially reduce prediction error for some models despite significant a priori bias. With two TDM measurements, MA achieved the lowest error (rRMSE, 26.9%; CCC, 0.971), followed by our developed model (rRMSE, 31.4%; CCC, 0.959) and most published models (rRMSE, 34.8%–36.1%). The Cojutti et al. model (33), however, retained substantial bias (MDPE, −26.0%; rRMSE, 66.3%), suggesting either inadequate structural specification or omission of critical covariates that routine TDM data cannot compensate for. This persistent underperformance confirms previously reported limitations (52). To our knowledge, this is the first application of MA to dalbavancin TDM. By weighting predictions, MA combines structural assumptions to mitigate single-model uncertainty. Despite inherent limitations (dependence on constituent model quality, reduced mechanistic interpretability, and undefined optimal weighting), MA provided a 14% relative improvement in prediction accuracy over the best single model. For precision dosing, where forecasting accuracy directly informs re-dosing timing, this incremental gain may reduce the risk of subtherapeutic exposure during prolonged therapy. However, validation in larger, more diverse cohorts remains necessary to assess generalizability across patient subgroups (37–39, 52–55).

Our PTA simulations indicate that standard two-dose regimens provide 29–48 days of coverage (MIC 0.25 mg/L), depending on eGFR and WT, with substantial interindividual variability (95% CI 22–91 days). Patients with high eGFR or high WT frequently lose target coverage earlier and may benefit from earlier or additional dosing. A third 1,500 mg dose, administered when PTA falls below 90%, largely restored extended coverage across all strata. Notably, heavier patients (≥80 kg) under D1/D15 may experience transient gaps in coverage before the day 15 dose when treating less susceptible pathogens (MIC 0.25 mg/L), suggesting D1/D8 may be more reliable for this subpopulation. These findings align with Baiardi et al. (34) and Cojutti et al. (35), though direct comparison is complicated by different PD targets and MIC assumptions. All studies converge on key findings: PTA duration is highly sensitive to the PD target, renal function, and body weight, with substantial interindividual variability consistently observed despite these covariate relationships.

The PK/PD target (*f*AUC_24_/MIC ≥ 111.1) reflects pre-clinical efficacy data (2-log10 bacterial reduction) and recent clinical observations in gram-positive infections (30, 31, 56, 57). Although validated PD targets for streptococci and enterococci are lacking, dalbavancin demonstrates potent *in vitro* activity against these organisms, with MIC_90_ values comparable to or even lower than those for *Staphylococcus aureus* (e.g., *Streptococcus* spp. MIC_90_ ≤0.03 mg/L and *E. faecalis* MIC_90_ ~0.03 to 0.06 mg/L) (15, 17, 58).

The substantial interindividual variability in PTA duration necessitates individualized dosing for prolonged therapy. Based on our pharmacokinetic findings, we propose an investigational sequential MIPD workflow. The first component (early detection trigger) would employ an ML algorithm (SVM) leveraging baseline covariates and a single pre-second-dose concentration to classify patient exposure on a weekly basis, identifying the exact week when target attainment is predicted to drop below the efficacy threshold. In our prior work (41), this approach demonstrated superior sensitivity for predicting the onset of subtherapeutic exposure compared to standard MAP-BE, achieving zero false negatives during external validation. The second component (longitudinal dose optimization) would shift to a mechanistic popPK approach once the ML algorithm triggers the need for a third dose. By incorporating the newly drawn pre-dose concentration, MAP-BE would update the individual pharmacokinetic profile to determine the precise dose and optimal interval for subsequent administrations. However, this integrated workflow has not been tested prospectively as a complete system. While individual components show promise, prospective validation should assess the accuracy of ML-triggered re-dosing timing vs empirical schedules and the impact on clinical outcomes (infection cure and relapse rates).

To facilitate future evaluation, we developed an open-source web application (https://sayadi-h.shinyapps.io/Optimizing_dalbavancin_dosing/) implementing this investigational approach (59). This application integrates both the ML and PK components into a unified interface. In the first module, clinicians can input baseline patient covariates alongside a single early dalbavancin concentration (day 8 or day 15) to execute the ML algorithm, immediately yielding an anticipated timeframe for target attainment failure. Once longitudinal TDM data become available, the second module performs MAP-BE. Crucially, the application automatically computes individualized PK profiles across all six evaluated popPK models and dynamically applies the MA to generate a consensus probability of target attainment. By automating advanced computational steps, this tool could eventually bridge the gap between complex pharmacometrics and bedside decision-making if validated, offering a scalable solution to logistical barriers limiting real-world Bayesian TDM implementation.

This study presents certain limitations. The retrospective design inherently risks selection bias toward complicated cases requiring intensive monitoring. Additionally, the modest external validation sample (*n* = 30, 92 concentrations) limits statistical power, precluding definitive claims of model superiority despite achieving the lowest a priori rRMSE and warranting broader validation across diverse subgroups. Serum albumin was not systematically available and could not be evaluated as a covariate, despite dalbavancin’s high protein binding. For this drug, characterized by restrictive elimination, hypoalbuminemia primarily alters total concentrations and total clearance while having a limited effect on unbound exposure, the relevant driver of PK/PD activity (60, 61). However, this distinction is critical for TDM interpretation: the clinical use of total concentration thresholds requires caution in patients with marked hypoalbuminemia, as total concentrations may falsely suggest subtherapeutic exposure despite adequate unbound levels, potentially leading to unnecessary dose escalation. Furthermore, sparse, late-phase sampling induced high shrinkage on distribution parameters, limiting peak concentration predictions. Nevertheless, moderate clearance shrinkage (27.7%) preserves the reliability of *f*AUC_24_, the primary PK/PD driver, ensuring robust TDM and PTA forecasting for prolonged therapy. Finally, lacking direct clinical outcomes (cure, relapse, and mortality), this analysis relies on PK/PD target attainment as a validated surrogate endpoint. Consequently, the proposed sequential ML/MAP-BE workflow and web application represent an investigational framework requiring prospective clinical validation prior to routine implementation.

### Conclusion

This study developed and externally validated a population pharmacokinetic model of dalbavancin in the largest real-world TDM cohort to date (*n* = 183), demonstrating through head-to-head benchmarking that model averaging, applied here for the first time to dalbavancin, achieves optimal predictive performance with routine TDM data. Monte Carlo simulations indicate that standard two-dose regimens maintain target attainment for 3–7 weeks, depending on renal function and body weight, with substantial interindividual variability necessitating TDM-guided individualization for extended therapy. The proposed sequential ML and MAP-BE workflow shows promise as an investigational framework but requires prospective clinical validation before implementation.

## Data Availability

The data supporting the findings of this study consist of pseudonymised patient health data and are subject to ethical, legal, and privacy restrictions. Therefore, the data are not publicly available and cannot be deposited in a public repository.

## SUPPLEMENTAL MATERIAL

Supplemental material aac.00665-26-s0001.docx Fig. S1 to S5; Table S1.

ASM does not own the copyrights to Supplemental Material that may be linked to, or accessed through, an article. The authors have granted ASM a non-exclusive, world-wide license to publish the Supplemental Material files. Please contact the corresponding author directly for reuse.
