# Supplementary material for: Population pharmacokinetics of dalbavancin: external validation, model averaging, and implications for precision dosing in prolonged therapy
Source: Antimicrob Agents Chemother. 2026 Jun 22;70(8):e00665-26. doi: 10.1128/aac.00665-26 (PMC13436002; doi:10.1128/aac.00665-26)
Supplement: Supplementary file 1 [file aac.00665-26-s0001.docx]

# Figure list:

Fig. S1. Individual fits.

Fig. S2. Diagnostic plots for model validation and adequacy assessment.

Fig. S3. Observed versus predicted dalbavancin concentrations in the external validation cohort — a priori predictions (no TDM information)

Fig. S4. Observed versus predicted dalbavancin concentrations in the external validation cohort — maximum a posteriori Bayesian estimation (MAP-BE) with one TDM measurement.

Fig. S5. Observed versus predicted dalbavancin concentrations in the external validation cohort — MAP-BE with two TDM measurements.

Fig. S1. Individual fits

Fig. S2. Diagnostic plots for model validation and adequacy assessment.

A) Numerical predictive check. Cumulative distribution of observed concentrations (red dots) compared with the theoretical distribution (blue line) and 95% prediction interval from 1,000 simulations (shaded area). The close alignment confirms adequate predictive performance of the population model across the entire concentration range.

(B) Empirical distributions of individual pharmacokinetic parameters. Histograms of individual Bayesian estimates (empirical Bayes estimates) for clearance (CL), central volume of distribution (V1), intercompartmental clearance (Q), and peripheral volume of distribution (V2). The empirical distributions closely follow the assumed log-normal distributions (blue curves).

(C) Standardized random effects (eta). Boxplots of individual deviations (eta) for CL, V1, Q, and V2, standardized to mean = 0 and variance = 1. Red dots represent individual patient values.

Fig. S3. Observed versus predicted dalbavancin concentrations in the external validation cohort — a priori predictions (no TDM information)

Population predictions based solely on patient covariates for six population pharmacokinetic models: developed model (this work), Carrothers et al., Cojutti et al. 2021, Cojutti et al. 2023, Chiriac et al., and Baiardi et al. Each panel represents one model. The solid black line indicates the line of identity (perfect prediction), dashed gray lines indicate the ±20% prediction interval, and the red line shows the linear regression fit. The relative root mean squared error (rRMSE, %) quantifies predictive performance normalized to the mean observed concentration. Detailed model specifications are provided in Table 1.

Fig. S4. Observed versus predicted dalbavancin concentrations in the external validation cohort — maximum a posteriori Bayesian estimation (MAP-BE) with one TDM measurement.

Individual Bayesian predictions incorporating one previous dalbavancin concentration for seven approaches: the six population pharmacokinetic models evaluated (developed model, Carrothers, Cojutti 2021, Cojutti 2023, Chiriac, Baiardi) plus a Bayesian model averaging approach (MA). The MA approach combines predictions from all six models weighted by individual log-likelihoods, following the methodology of Uster et al. Each panel represents one model or approach. The solid black line indicates the line of identity, dashed gray lines indicate the ±20% prediction interval, and the red line shows the linear regression fit. rRMSE values (%) quantify predictive performance.

Fig. S5. Observed versus predicted dalbavancin concentrations in the external validation cohort — MAP-BE with two TDM measurements.

Individual Bayesian predictions incorporating two previous dalbavancin concentrations for the same seven approaches as Figure S4. Each panel represents one model or approach. The solid black line indicates the line of identity, dashed gray lines indicate the ±20% prediction interval, and the red line shows the linear regression fit. rRMSE values (%) quantify predictive performance. Model averaging demonstrated optimal performance in this scenario (rRMSE 26.9%; CCC 0.971), as shown in Table 5.

# Table list:

Table S1. Duration of optimal PTA (≥90%) following dalbavancin dosing regimens stratified by eGFR and body weight

Table S1. Duration of optimal PTA (≥90%) following dalbavancin dosing regimens stratified by eGFR and body weight

| **Regimen** | **Covariate** | | **Stratum** | **3rd dose: MIC 0.125 (day [95% CI])** | | **3rd dose: MIC 0.250 (day [95% CI])** | **4th dose: MIC 0.125 (day [95% CI])** | **4th dose: MIC 0.250 (day [95% CI])** | |
| --- | --- | --- | --- | --- | --- | --- | --- | --- | --- |
| D1/D15 | | Weight | 120-200 | | 34 [30–82] | 26 [24–52] | 57 [51–100] | | 40 [37–75] |
| D1/D15 | | Weight | 30-49 | | 50 [44–96] | 41 [36–74] | 83 [78–100] | | 66 [62–101] |
| D1/D15 | | Weight | 50-79 | | 50 [44–96] | 37 [30–69] | 83 [78–100] | | 60 [55–96] |
| D1/D15 | | Weight | 80-119 | | 45 [35–91] | 29 [26–62] | 76 [68–100] | | 48 [43–87] |
| D1/D15 | | eGFR | 15-29 | | 64 [56–100] | 48 [41–91] | 100 [100–100] | | 79 [73–100] |
| D1/D15 | | eGFR | 30-59 | | 56 [49–10O] | 41 [34–78] | 94 [88–100] | | 68 [62–100] |
| D1/D15 | | eGFR | 60-89 | | 51 [44–96] | 36 [30–70] | 85 [78–100] | | 60 [53–97] |
| D1/D15 | | eGFR | 90-120 | | 47 [40–88] | 34 [28–64] | 78 [73–100] | | 55 [50–90] |
| D1/D8 | | Weight | 120-200 | | 29 [24–76] | 20 [18–49] | 52 [46–100] | | 34 [31–70] |
| D1/D8 | | Weight | 30-49 | | 45 [38–91] | 36 [31–70] | 78 [73–100] | | 61 [57–97] |
| D1/D8 | | Weight | 50-79 | | 45 [39–90] | 32 [25–66] | 79 [73–100] | | 56 [50–92] |
| D1/D8 | | Weight | 80-119 | | 40 [30–86] | 24 [20–59] | 71 [62–100] | | 43 [38–82] |
| D1/D8 | | eGFR | 15-29 | | 59 [51–100] | 44 [35–87] | 100 [96–100] | | 76 [69–100] |
| D1/D8 | | eGFR | 30-59 | | 51 [44–100] | 36 [28–75] | 89 [82–100] | | 63 [56–100] |
| D1/D8 | | eGFR | 60-89 | | 46 [39–93] | 32 [24–66] | 80 [73–100] | | 55 [49–93] |
| D1/D8 | | eGFR | 90-120 | | 43 [36–86] | 29 [22–61] | 74 [68–100] | | 50 [45–86] |

Values represent the day at which PTA <90% was reached (mean [95% CI]). The 95% confidence interval was calculated from the 2.5th and 97.5th percentiles of individual critical days across 10,000 simulated patients per stratum. MIC values: 0.125 mg/L (EUCAST breakpoint for Staphylococcus aureus) and 0.250 mg/L (EUCAST breakpoint for Enterococcus faecium). PTA (probability of target attainment) defined as fAUC₂₄/MIC ≥111.1. eGFR expressed in mL/min/1.73 m²; Weight in kg. D1/D8: 1,500 mg on Day 1 and Day 8. D1/D15: 1,500 mg on Day 1 and Day 15.

Predictive performance metrics:

Predictive performance of the models was quantified by comparing individual predictions (PRED or IPRED) to the observed concentrations (DV). For each observation i, the prediction error (PEi), the relative percentage error (rPEi), and the absolute percentage error (APEi) were calculated as follows:

$$PE\_i=\mathrm{PRE}D\_i-DV\_i$$

$$\mathrm{rP}E_{i} = \frac{\mathrm{PRE}D_{i} - DV_{i}}{DV_{i}} \times100$$

$$\mathrm{AP}E_{i}=\left| rPE_{i} \right|$$

Overall model performance was assessed using the following metrics:

1. Bias and precision:

- Median Prediction Error (MDPE): Evaluates the systematic bias of the model.

$$MDPE=median\left( rPE \right)$$

- Median Absolute Percentage Error (MDAPE): Evaluates the overall precision of the model.

$$MDAPE=median\left( APE \right)$$

- (The interquartile ranges [IQR] for both MDPE and MDAPE were also reported to reflect error dispersion).

2. Global error:

- Root Mean Square Error (RMSE):

$$RMSE=\sqrt{\left( 1/n\sum\left( i=1 \right)^{n}\left( PE_{i} \right)^{2} \right)}$$

- Relative RMSE (rRMSE): Scaled to the mean of the observed concentrations.

$$rRMSE=\left( RMSE/\bar{\left( DV \right)} \right)\times100$$

3. Clinical acceptability:

- f20 and f30: The proportion of predictions falling within ±20% and ±30% of the observed concentrations, respectively.
  - f20 = % of APE_i ≤ 20%
  - f30 = % of APE_i ≤ 30%

4. Concordance:

- Lin’s Concordance Correlation Coefficient (CCC): Evaluates the degree of agreement between predicted and observed values, factoring in both correlation (R²) and the bias correction factor (Cb).

Model averaging calculation:

For the Model Averaging (MA) approach, individual concentration predictions were computed in three sequential steps, following the methodology described by Uster et al.

First, individual MAP-BE estimation was performed independently for each of the six pharmacokinetic models using all available observed TDM concentrations for each patient.

Second, model-specific weights (wᵢ) were calculated based on individual log-likelihoods derived from each model's objective function value (OFV), assigning proportionally higher weights to models providing better individual fit:

$$w_{i}=exp\left( -0.5\times OFV_{i} \right)/\left( \sum_{j} exp\left( -0.5\times OFV_{j} \right) \right)$$

where OFVᵢ is the individual objective function value of model i, and the denominator sums over all six models. The weights satisfy Σᵢ wᵢ = 1.

Third, the final predicted concentration at each time point was computed as the weighted average of the individual posterior predictions from each constituent model:

$$\hat{\left( y \right)_{M}}A=\sum_{i} w_{i}\times\hat{\left( y \right)_{i}}$$

where ŷᵢ is the individual posterior prediction from model i.
